# Testicular Function of Childhood Cancer Survivors: Who Is Worse?

**DOI:** 10.3390/jcm8122204

**Published:** 2019-12-13

**Authors:** Ylenia Duca, Andrea Di Cataldo, Giovanna Russo, Emanuela Cannata, Giovanni Burgio, Michele Compagnone, Angela Alamo, Rosita A. Condorelli, Sandro La Vignera, Aldo E. Calogero

**Affiliations:** 1 Andrology and Endocrinology Unit, Department of Clinical and Experimental Medicine, University of Catania, 95123 Catania, Italy; ylenia.duca@gmail.com (Y.D.); burgio.giovanni88@libero.it (G.B.); michele.compagnone22@tiscali.it (M.C.); angela.alamo1986@gmail.com (A.A.); rosita.condorelli@unict.it (R.A.C.); acaloger@unict.it (A.E.C.); 2 Pediatric Oncohematology Unit, Department of Clinical and Experimental Medicine, University of Catania, 95123 Catania, Italy; adicata@unict.it (A.D.C.); diberuss@unict.it (G.R.); e.cannata80@gmail.com (E.C.)

**Keywords:** childhood cancer survivors, infertility, hypogonadism, testicular volume, azoospermia, chemotherapy, radiotherapy, stem cell transplantation, lymphoma, leukemia

## Abstract

Background: A multi-disciplinary approach has led to an improvement in prognosis of childhood cancers. However, in parallel with the increase in survival rate, there is a greater occurrence of long-term toxicity related to antineoplastic treatment. Hypogonadism and infertility are among the most frequent endocrinological sequelae in young adult childhood cancer survivors. The aim of this study was to identify which category of patients, grouped according to diagnosis, therapy, and age at treatment, shows the worst reproductive function in adulthood. Methods: We evaluated morpho-volumetric development of the testis, endocrine function of the hypothalamic–pituitary–gonadal axis, and sperm parameters in 102 young adult childhood cancer survivors. Results: Overall, about one-third of patients showed low total testicular volume, total testosterone (TT) <3.5 ng/mL, and altered sperm count. Hodgkin’s disease, hematopoietic stem cell transplantation, and non-cranial irradiation associated to chemotherapy were risk factors for poor gonadal function. Patients treated in pubertal age showed lower total testicular volume; however, the difference was due to more gonadotoxic treatment performed in older age. Testicular volume was more predictive of spermatogenesis than follicle-stimulating hormone (FSH), while anti-Müllerian hormone (AMH) was not useful in the evaluation of testicular function of male childhood cancer survivors. Conclusions: Pre-pubertal subjects at high risk of future infertility should be candidates for testicular tissue cryopreservation.

## 1. Introduction

Cancer in childhood and adolescence represents a rare pathology; however, it has a great biological interest and an extreme social and public health importance. A multi-disciplinary approach has led to an evident improvement in prognosis and quality of life for these patients who, more and more frequently, reach adulthood. Indeed, the drafting of increasingly effective diagnostic-therapeutic protocols has allowed to increase the 5-year survival rate from the 58% recorded at the end of the 1980s to around the 80% [1]. Therefore, in parallel with the increase in the number of children and adolescents who reach adulthood after having undergone treatment for cancer, there is a greater occurrence of long-term toxicities related to therapies carried out in pediatric age. 

Endocrine disorders are highly prevalent among cancer survivors; it has been estimated that 40–50% of survivors will develop at least one endocrinopathy over the course of their lifetime [2]. Among the negative endocrine long-term effects of antineoplastic treatments, gonadal failure and subsequent subfertility/infertility is one of the most frequent and most important for its economic and psycho-social implications. 

The gonadotoxic potential of some treatments, such as alkylating agents and testicular irradiation, is well known [3]. Chemotherapy-induced testicular damage is drug-specific and dose-related: the extent of the damage and the speed of recovery depend on the agent used and the dose received. Similarly, radiotherapy damages testicular germinal cells in a dose-dependent manner. However, not all children who undergo the same antineoplastic treatments will become infertile because individual susceptibility plays an important role in conditioning reproductive outcomes. Since adult testicular function is unpredictable, all boys already capable of ejaculating should, as a precaution, cryopreserve sperm before undergoing antineoplastic treatments. Pre-pubertal patients who do not yet have spermarche represent the category with major concerns about fertility, because the cryopreservation of testicular tissue with subsequently auto-graft is still an experimental procedure performed by few centers and with low probability of success [3]. 

Another issue regards the age at treatment. In past decades, it has been reported that patients treated at pre-pubertal age were protected from chemotherapy-induced testicular damage [4,5]. Authors hypothesized that the protective effect of very-young age was due to the relative quiescence of the germinal epithelium in the early stages of life [4,5]. Other research questioned this assumption [6,7,8,9], but recent studies are lacking.

In this study, we evaluated the endocrine function of the hypothalamic-pituitary-gonadal axis, the morpho-volumetric development of the testis, and sperm production in young adult men who underwent chemotherapy, irradiation, and/or bone marrow transplantation for the treatment of childhood cancer. The aim was to identify which, among these treatments, has the worst effect on testicular function in adulthood, for establishing which categories of childhood cancer patients should be recommended for testicular tissue cryopreservation. We also evaluated the influence of diagnosis and age at treatment on future fertility.

## 2. Materials and Methods

### 2.1. Study Population

This study involved 102 male long-term survivors of childhood cancer who underwent annual outpatient visits at the Pediatric Oncohematology Unit of the University Hospital “G. Rodolico” in Catania, Italy, between March 2018 and July 2019.

We enrolled patients aged between 16 and 40 years at the time of the visit, who had been treated with chemotherapy only, chemotherapy plus radiotherapy, and/or bone marrow transplantation and who were declared disease-free for at least 5 years. Patients treated with surgery only, testicular irradiation or orchiectomy for testicular neoplasia, and/or who were in testosterone replacement therapy at the time of the visit were excluded.

Each patient underwent a thorough health assessment including medical history collection, general and andrological physical examination, and scrotal ultrasound. Most patients underwent blood sampling for the dosage of luteinizing hormone (LH), follicle-stimulating hormone (FSH), and total testosterone (TT). A part of the patients underwent also anti-Müllerian hormone (AMH) dosage. Patients who accepted to undergo sperm analysis provided a semen sample. 

### 2.2. Scrotal Ultrasound

All patients underwent testicular ultrasound, performed by the medical staff of the Andrology and Endocrinology Unit of the University Hospital “Gaspare Rodolico” (Catania, Italy).

By ultrasound, we evaluated testicular volume, echotexture, and echo structure. Total testicular volume, resulting from the sum of the volume of the two testes, was considered normal when ≥24 mL. Echo structure and texture were considered normal if they were normoechoic and homogeneous.

By ecocolor Doppler, performed in basal conditions and during the Valsalva maneuver, it was also possible to explore the presence of varicocele and to assess its grading according to the Sarteschi classification. Varicocele was considered clinically significant in presence of basal, uni- or bilateral, venous reflux (grade IV Sarteschi).

Ultrasound scrotal evaluation was performed through an Esaote Mylab25 ultrasound scanner with a 7.5 MHz linear probe.

### 2.3. Hormone Essays

The measurement of LH, FSH, and TT was performed on blood samples by electrochemiluminescence (ECLIA). AMH was evaluated by one-step simultaneous enzyme immunoassay (“sandwich”).

Blood was collected at the same time as the oncohematological outpatient visit, that may occur in the morning or in the afternoon. For this reason, fasting was not required.

### 2.4. Semen Analysis

Patients were asked to deliver a semen sample by masturbation after a sexual abstinence of 3–5 days. The collection was carried out in a dedicated room adjacent to the seminology laboratory of the Andrology and Endocrinology Unit, within a sterile, wide-mouthed plastic container. The samples were analyzed by a qualified medical operator soon after liquefaction and, in any case, within an hour after ejaculation. The semen analysis was performed in accordance with the 2010 World Health Organization manual (5th edition) [10].

Semen samples with no visible sperm were centrifuged at 3000× *g* for 10 min. If no sperms were found after examination of 40 microscopic fields of the sediment at 400× magnification, the sample was considered azoospermic.

### 2.5. Statistical Analysis

The data obtained were analyzed with PHStat and RealStatistics add-on for Excel and all results were expressed as mean ± SD. The Shapiro–Wilk test was used to establish the normality of the distribution. Data with normal distribution was compared with Student’s *t*-test; while non-parametric data was examined with the Mann–Whitney U test. Pearson’s correlation coefficient was used to assess correlations between sperm concentration, testicular volume, and FSH. Simple and multivariate regression was performed for testicular volume. One-tailed *p* ≤ 0.05 was considered significant.

### 2.6. Ethical Approval

The protocol was approved by the internal Institutional Review Board (ethical approval code: 27/2018), and informed consent was obtained from each participant after full explanation of the purpose and nature of all procedures used. The study was conducted in accordance with the principles expressed in the Declaration of Helsinki.

All patients were made aware of the results of their clinical tests and who showed hormonal, ultrasound, and/or seminal alterations was included in a clinical and instrumental monitoring program at the Andrology and Endocrinology Unit of the University Hospital of Catania.

## 3. Results

**Figure 1:**
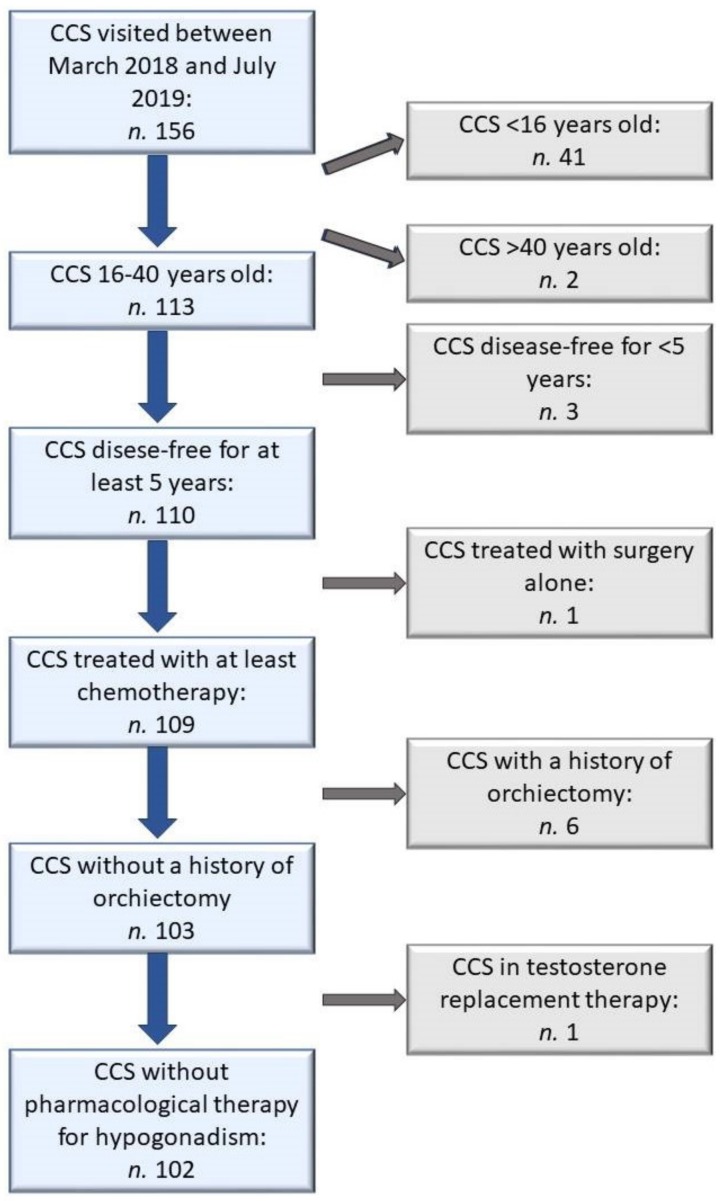
Flow-chart of sample selection process. CCS: Childhood cancer survivors.

One hundred and fifty-six male patients visited the outpatient clinic dedicated to long-term survivors of childhood cancer between March 2018 and July 2019. Among them, 54 patients were excluded: 41 were too young, 2 were too old, 3 had finished antineoplastic treatment less than five years before, 1 was treated with surgery alone, 6 underwent unilateral orchiectomy for testicular tumor, 1 was on testosterone replacement therapy for an already diagnosed primary hypogonadism (Figure 1).

**Figure 2:**
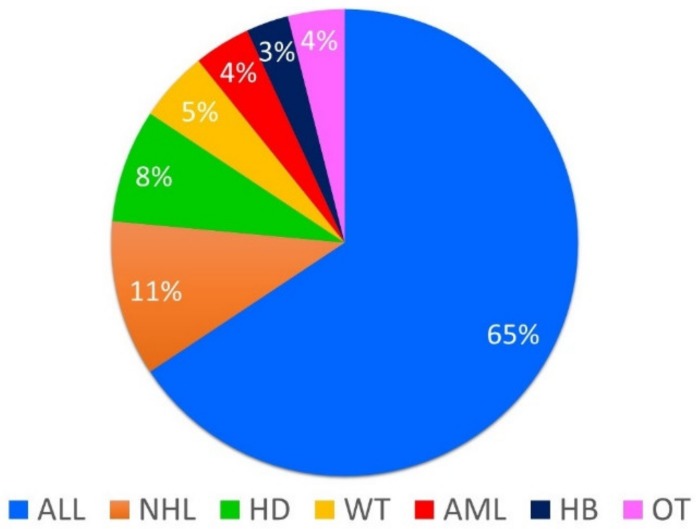
Frequency of diagnosis in 102 childhood cancer survivors. ALL: acute lymphoblastic leukemia; AML: acute myeloid leukemia; HB: hepatoblastoma; HD: Hodgkin’s disease; NHL: non-Hodgkin lymphoma; OT: other malignancies (histiocytosis, nasopharyngeal carcinoma, rhabdomyosarcoma, and Ewing’s sarcoma); WT: Wilms tumor.

A total of 102 patients, aged 16–38 years, were enrolled. Mean age at diagnosis was 6.2 ± 4.2 years; mean age at enrollment was 23.2 ± 5.4 years (Table 1). Main diagnoses were acute lymphoblastic leukemia (ALL) (*n*. 67), non-Hodgkin’s lymphoma (NHL) (*n*. 11), Hodgkin’s disease (HD) (*n*. 8), Wilms tumor (WT) (*n*. 5), acute myeloid leukemia (AML) (*n*. 4), and hepatoblastoma (HB) (*n*. 3). The other 4 patients had been affected by histiocytosis, nasopharyngeal carcinoma, rhabdomyosarcoma, and Ewing’s sarcoma (Figure 2). All patients had been treated with at least chemotherapy, according to the protocols of Italian Association of Pediatric Hematology and Oncology (AIEOP), based on the type and stage of the malignancy. In addition to chemotherapy, 28 patients also received irradiation and 5 underwent hematopoietic stem cell transplantation (SCT).

### 3.1. Grouping According to Diagnosis, Therapy, and Age of Treatment

Childhood cancer survivors were allocated to five categories according to diagnosis: ALL, NHL, HD, WT, and others (OT). The OT group included all diagnosis which occurred in less than 5 patients (acute myeloid leukemia, hepatoblastoma, histiocytosis, nasopharyngeal carcinoma, rhabdomyosarcoma, and Ewing’s sarcoma).

Patients were also classified according to the treatment received: chemotherapy only (CO), chemotherapy plus radiotherapy (CR), hematopoietic stem cell transplantation (SCT). The CR group was further divided into two subgroups: chemotherapy plus cranial irradiation (CRc) and chemotherapy plus non-cranial irradiation (CRn).

According to the age of treatment, patients were subdivided into two group: patients treated in prepubertal age (˂10 years old) and patients treated in pubertal age (≥10 years old). The cut-off of 10 years was chosen based on the report of Nielsen and colleagues indicating the age of 11.7 years as the lower range of the interval at which spermarche occurs. Thus, under the age of 10, all subjects were probably pre-pubertal [11]. The same cut-off was previously used in other studies [12,13].

### 3.2. Testicular Morpho-Volumetry

All enrolled childhood cancer survivors underwent scrotal ultrasound. Mean right, left, and total testicular volume were in the normal range (Table 1). However, total testicular volume was low (˂24 mL) in one third of patients (*n*. 38). Twenty out of 102 patients (19.6%) had a clinically significant varicocele (≥IV grade of Sarteschi classification). 

**Figure 3:**
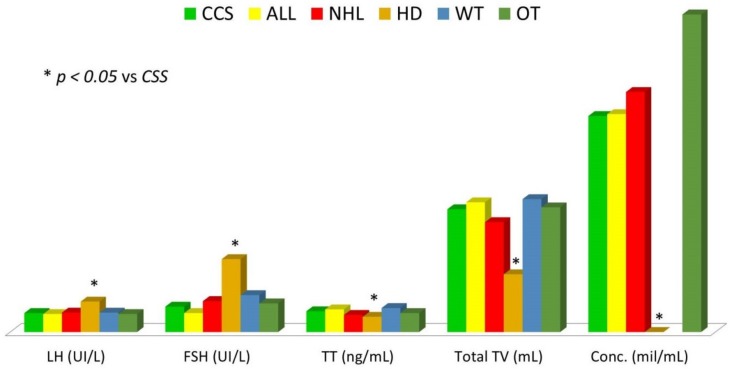
Comparison of hormonal values, total testicular volume, and sperm concentration among childhood cancer survivors grouped according to diagnosis. ALL: acute lymphoblastic leukemia; CCS: childhood cancer survivors; Conc.: sperm concentration; FSH: follicle-stimulating hormone; HD: Hodgkin’s disease; LH: luteinizing hormone; NHL: non-Hodgkin lymphoma; TT: total testosterone; TV: testicular volume; OT: other malignancies; WT: Wilms tumor.

After patients’ grouping according to diagnosis, only the HD group showed significantly lower total testicular volume values compared to the overall sample of childhood cancer survivors (*p* ˂ 0.001) (Table 2, Figure 3).

**Figure 4:**
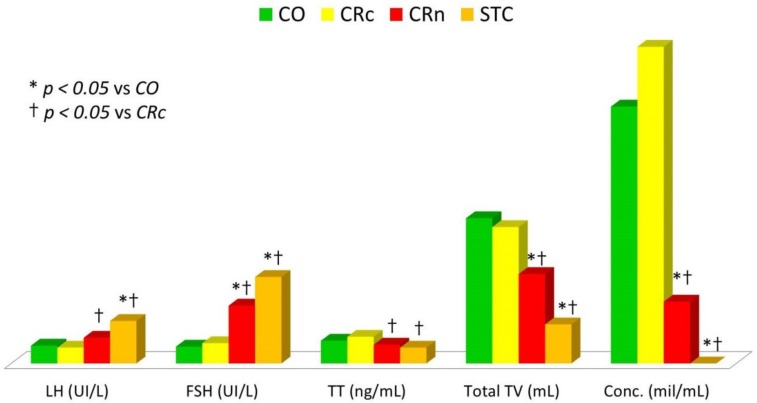
Comparison of hormonal values, total testicular volume, and sperm concentration among childhood cancer survivors grouped according to therapy. CO: chemotherapy only; Conc.: sperm concentration; CRc: chemotherapy plus cranial irradiation; CRn: chemotherapy plus non-cranial irradiation; FSH: follicle-stimulating hormone; LH: luteinizing hormone; STC: stem cell transplantation; TT: total testosterone; TV: testicular volume.

After grouping according to therapy, the CO group showed significantly higher total testicular volume compared to overall sample of childhood cancer survivors (*p* = 0.015); the CR group had significantly lower total testicular volume than the CO group (*p* ˂ 0.001); the SCT group showed significantly lower total testicular volume than both CO (*p* ˂ 0.001) and CR groups (*p* = 0.001). After further subdividing patients belonging to the CR group in two different categories according to the irradiated site (cranial or non-cranial), total testicular volume of CRc patients showed no statistically significant differences compared to the CO group. On the contrary, the CRn group had significantly lower total testicular volume compared to both CO (*p* ˂ 0.001) and CRc (*p* = 0.002) groups (Table 1, Figure 4).

After subdivision for age of treatment, patients treated when they were ≥10 years old showed slightly but significantly lower total testicular volume compared to patient aged ˂10 years at the time of treatment (*p* = 0.046) (Table 1). However, data became not significant when adjusted for diagnosis and treatment. In patients treated with chemotherapy only (CO), there was a trend for a lower testicular volume in survivors treated in pubertal age, but the difference did not reach statistical significance (*p* = 0.052). To confirm our assumption, we built a multivariate regression model, including age at the time of treatment, diagnosis, modality of treatment, and presence of varicocele. With backward procedure, we were able to establish that therapy was the only variable significantly related to testicular volume, alone explaining 36% of the volume variability.

### 3.3. Endocrine Function of the Hypothalamic–Pituitary–Testis Axis

Eighty-seven childhood cancers survivors underwent hormonal evaluation. Overall mean hormonal values were in the normal range (Table 1). Three out of 87 patients (3.4%) and 5/87 patients (5.7%) exhibited respectively below or above normal LH levels. None of the patients had reduced FSH values, while 8/87 patients (9.2%) showed FSH levels above normal range. Twenty eight of the 84 patients (33.3%) had testosterone values ˂3.5 ng/mL; however, only 5 patients (6%) showed frankly reduced TT levels (˂2.3 ng/mL). Testosterone values of three patients were missing because of lab errors. 

Of blood samples from 17 patients, we also evaluated AMH concentrations. None of the childhood cancers survivors had low AMH levels, while only 1 patient had AMH values above normal. 

After patients’ grouping according to diagnosis, only the HD group showed significantly higher LH and FSH values and significantly lower TT values compared to overall sample of childhood cancer survivors (*p* = 0.013, ˂0.001, and 0.027, respectively) (Table 2, Figure 3).

After grouping according to therapy, the CO group showed slightly but significantly lower FSH values compared to the overall sample of childhood cancer survivors (*p* = 0.047); the CR group had significantly higher FSH values than the CO group (*p* = 0.009); the SCT group showed significantly higher FSH and LH values compared to both CO (*p* = 0.001 and *p* ˂ 0.001) and CR groups (*p* = 0.004 and *p* = 0.008). After further subdividing the CR group patients in two different categories according to the irradiated site (cranial or non-cranial), the CRc group showed hormonal values comparable to the CO group, except for TT values that were slightly but significantly higher (*p* = 0.042). On the contrary, the CRn group had significantly higher FSH levels than both CO (*p* ˂ 0.001) and CRc (*p* = 0.001) groups, and higher LH and lower TT values than the CRc group (*p* = 0.02 and 0.003, respectively) (Table 1, Figure 4).

After subdivision for age of treatment, the two groups (˂10 years old and ≥10 years old) showed no significant differences in hormone levels (Table 1).

### 3.4. Sperm Parameters

Thirty-four patients accepted to undergo sperm analysis. Overall mean sperm concentration and count were in the normal range (Table 1). Sperm count was reduced in 7/34 patients (20.6%), while azoospermia was found in 6/34 patients (17.7%). Overall, 38.2% of patients showed low or absent sperm production. All azoospermic patients had primary testicular disease, characterized by FSH levels >8 UI/L and low testicular volume, and had undergone radiotherapy or SCT. Among oligozoospermic patients, 5 had normal FSH values and testicular volume and 2 had normal FSH values and slightly reduced testicular volume. Four of them showed clinically significant varicocele.

After patients’ grouping according to diagnosis, only the HD group showed significantly lower sperm concentration compared to the overall sample of childhood cancer survivors (*p* = 0.029) (Table 2, Figure 3). 

After grouping according to therapy, only the SCT group showed reduced sperm concentration compared to the CO group (*p* = 0.004) and to the overall sample of childhood cancer survivors (*p* = 0.025). When the CR group was further subdivided into two categories according to the irradiated site, the CRn group showed lower sperm concentration than both CO and CRc groups (*p* = 0.007 and 0.036, respectively) (Table 1, Figure 4).

After subdivision for age of treatment, there was no significant difference between the two groups (˂10 years old and ≥10 years old) in terms of sperm concentration (Table 1).

Pearson’s correlation coefficient revealed that sperm concentration was related more to testicular volume (ρ = 0.51, *p* = 0.002) than FSH (ρ = –0.43, *p* = 0.01).

## 4. Discussion

Only one third of patients accepted to undergo sperm analysis. Many childhood cancer survivors refused saying they did not want to know anything about their reproductive function or that they were not willing to have children, now or in the future. The same reluctance was described by other authors [12,14,15]. This common attitude among childhood cancer survivors has led authors to look for surrogate markers of spermatogenesis to be used in infertility screening. Inhibin B has proved to be a better marker for spermatogenesis than FSH [12,14]. However, in some studies, testicular size was shown to be a predictor of sperm production even better than serum inhibin B [16]. The difference in the predictive power of testicular volume depends on the applied measurement methods. Most of the studies that did not show statistically significant differences between testicular volume of patients and controls used the orchidometer [12]. To our knowledge, ours is one of the few studies about childhood cancer survivors in which testicular volume has been measured by ultrasound. This is a strength, since it has been shown that orchidometer is less accurate than ultrasound in volume evaluation, especially in testes smaller than 18 mL [17]. By ultrasound, we showed that testicular volume is a predictor of poor spermatogenesis better than FSH, but we were not able to compare it with inhibin B since we could not measure this hormone. AMH showed no usefulness in gonadal evaluation of young adult male long-term childhood cancer survivors. The only patient with elevated AMH levels was 22 years old and he was treated for NHL at 3 years of age with chemotherapy only. He exhibited normal gonadotropin levels, TT levels in the “grey zone”, and modestly decreased total testicular volume, but he refused to undergo sperm analysis. 

In this study we found a high percentage of patients (one third) with suboptimal TT levels; however, a low prevalence of overt hypogonadism (TT ≤2.3 ng/mL) and altered LH levels were recorded. This probably occurred because blood samples for hormone essays were not drawn in standardized condition. According to Endocrine Society guidelines, testosterone must be measured in the morning after 8 h of fasting and the diagnosis must be confirmed repeating the blood sampling a second time under the same conditions [18]. We were unable to follow these indications because, to improve patients’ compliance, we performed blood sampling at the same time of the outpatient visit, which sometimes took place also in the afternoon.

In the past decades, it has been hypothesized that pre-pubertal testis is less sensitive than post-pubertal testis to chemotherapy-induced damage. Some reports supported this assumption [4,5], while other studies did not [6,7,8,9]. The Childhood Cancer Survivor Study reported that only boys ˂4 years of age at diagnosis were more likely to sire a pregnancy later in life than those who were 15–20 years of age at diagnosis [19]. In our study, the only parameter marginally related to the age at diagnosis was testicular volume, but a more careful analysis found that the difference was due to therapy: in pubertal and post-pubertal age, HD diagnosis and combined therapy with alkylating drugs and irradiation were more frequent, while in early childhood LLA and WT were the most common diagnosis, requiring less gonadotoxic therapy.

Data from the Childhood Cancer Survivor Study showed that the main risk factors for male infertility after antineoplastic treatment were: diagnosis of HD, testicular irradiation with ≥7.5 Gy, and alkylating agent dose score ≥2; while pituitary irradiation was not related to impaired fertility [19]. Our study confirmed that cranial irradiation with up to 24 Gy does not affect pituitary gonadotropin secretion as shown by the fact that childhood cancer survivors who underwent chemotherapy and cranial irradiation showed similar hormone levels than patients treated with only chemotherapy. None of our patients received cranial radiation dose >24 Gy. 

Unfortunately, we have not been able to subdivide patients according to alkylating agent dose score because for many of them (especially the older ones), it was not possible to trace the exact dose of chemotherapy received. Thus, we preferred to divide patients according to the diagnosis, assuming that patients suffering from the same pathology had received similar treatment schemes and could represent a homogeneous group.

Similar to the results of the Childhood Cancer Survivor Study, we found that HD was the diagnosis with the worst testicular function. HD patients usually undergo higher doses of alkylating and alkylating-like agents; however, they routinely also undergo thoracic and/or abdominal irradiation. It is known that not only direct testicular irradiation but also scattered dose could be responsible for testicular damage [20]. We hypothesize that testicular damage in HD patients could be partially due to scattered testicular irradiation. Our assumption is supported by the finding that childhood cancers survivors who underwent non-cranial irradiation have poorer testicular function than both patients who underwent chemotherapy only and chemotherapy plus cranial irradiation, independently from diagnosis. Indeed, the CRn group also included patients who underwent non-cranial irradiation for other malignancies than HD (nasopharyngeal carcinoma, rhabdomyosarcoma, Wilms tumor, Ewing’s sarcoma). 

As expected, patients who underwent HSCT had poor testicular function: all of them had low testicular volume and all of them except one had high FSH levels. Among them, the two patients who accepted to perform semen analysis were azoospermic.

Currently, fertility preservation treatments for pre-pubertal patients who do not yet produce mature spermatozoa are lacking. Some centers have started to cryopreserve immature testicular tissue from pre-pubertal boys before starting chemotherapy treatment. In 2015, seven centers in Europe had already collected and stored more than 260 pre-pubertal testicular tissue samples, with biopsies undertaken only when treatment was deemed at high risk for later fertility complications [3]. It is not yet certain if such cryopreserved tissue can be successfully used later to restore fertility in humans. However, the generation of sperm-like cells after 3D culture of isolated spermatogonial cells obtained from testis biopsies taken from pre-pubertal boys undergoing chemotherapy treatment has been recently described [21]. In a murine model, complete spermatogenesis was achieved through the in vitro culture of spermatogonial germ cells; the obtained mature spermatozoa have then been used to produce viable embryos through in vitro fertilization/ intracytoplasmic sperm injection (IVF/ICSI) [22]. Another frontier of fertility preservation in male childhood cancer survivors is the transplantation of spermatogonial stem cells or frozen-thawed immature testicular tissue grafted back into the adult. This technique has been successful in producing functional gametes in animal models, including non-human primates and mice, and healthy offspring have been produced through IVF/ICSI using sperm derived from xenografted immature testicular tissue. The concerns are about the possible reintroduction of malignant cells in case of unapparent leukemic infiltration of the xenografted tissue [3]. 

Instead of trying to restore fertility, an alternative strategy would be the development of interventions aimed at preventing chemotherapy- or radiation-induced testicular damage. Cytoprotective agents that specifically protect the pre-pubertal testis without interfering with the toxicity to cancer cells could potentially be employed as part of the treatment regimen. In vivo animal studies investigated the effects of the administration of antioxidants (amifostine, carnitine, ginseng intestinal metabolite I, vitamin C, and curcumin) prior to chemotherapy reporting contrasting results [3]. 

In post pubertal boys and men, a prophylactic down-regulation of the pituitary gland by gonadotropin-releasing hormone (GnRH) agonists or testosterone to induce a quiescent state in the gonads was proposed. This approach was attempted for the belief that the prepubescent testis was less sensitive to gonadal toxicity than the active adult one, as demonstrated by old studies [5]. However, more recent data—including ours—showed that both pre-pubertal and post-pubertal testis are sensible to the gonadotoxic damage of chemo- and radiotherapy [23]. 

Regarding radioprotection techniques, modern shielding systems allow to reduce the scattered radiation to the testis up to 1% of the prescribed dose. However, the received dose depends primarily on the distance from the field edge to the gonads [24]. Modifications in irradiation techniques has reduced radiation dose scattered to testis during treatment of pelvic fields. For example, proton radiotherapy significantly reduced scattered dose to normal tissues compared with intensity-modulated radiotherapy in male patients treated for pediatric pelvic rhabdomyosarcoma [25]. This could suggest a wider use of proton radiotherapy instead of intensity-modulated radiotherapy also in other pediatric malignancies in order to better safeguard future reproductive function; however, further studies are needed.

## 5. Conclusions

Our study showed that patients with the worst reproductive function among childhood cancers survivors are those with previous diagnosis of HD and treated with non-cranial irradiation in addition to chemotherapy and/or STC. Patients treated with chemotherapy only and chemotherapy plus cranial irradiation did not show, as a group, any impairment of testicular function. Age at diagnosis (pre-pubertal or pubertal) does not also seem to influence gonadal function of childhood cancer survivors in young adulthood. However, generalizations cannot always be applied to the specific case. 

Indeed, due to individual susceptibility to chemotherapy- and radiotherapy-induced testicular damage, the resumption of spermatogenesis after different kinds of treatments tends to be unpredictable, and studies on spermatogenesis in long-term cancer survivors have shown evidence of persistent azoospermia or severe oligozoospermia in up to 24% of patients [13,26]. For these reasons, sperm cryopreservation—if possible—is always indicated before starting antineoplastic treatment. In 20% of Tanner II boys, spermiogenesis has already started, allowing cryopreservation of sperm from that age. Thus, pubertal boys with testicular volumes greater than 10–12 mL should give semen samples before undergoing antineoplastic therapy [20].

Finally, our study showed that testicular volume evaluated by ultrasound is more predictive of spermatogenesis than FSH. Our preliminary data also indicate that AMH is not useful in the evaluation of testicular function in childhood cancer survivors. 

## Figures and Tables

**Table 1:** Clinical features of childhood cancer survivors, overall and according to therapy and age at the time of treatment (mean ± DS). AMH: anti-Müllerian hormone: CCS: childhood cancer; CO: chemotherapy only; CRc: chemotherapy plus cranial irradiation; CRn: chemotherapy plus non-cranial irradiation; FSH: follicle-stimulating hormone; LH: luteinizing hormone; STC: stem cell transplantation; TT: total testosterone; TV: testicular volume. Reference values: TV ≥ 24 mL, LH 1.14–8.75 UI/L, FSH 0.95–11.95 UI/L, TT ≥3.5 ng/mL (gray zone 2.3–3.5 ng/mL), AMH 0.73–16.05 ng/mL, sperm concentration >39 × 10^6^/mL.

Clinical Features	All CSS	CO	CRc	CRn	STC	˂10 Years	≥10 Years
Age at diagnosis (years)	6.2 ± 4.2	5.7 ± 3.8	6.5 ± 5.1	8.2 ± 4.2	6.6 ± 5.2	4.2 ± 2.3	12.3 ± 2.0
Age at enrollment (years)	23.2 ± 5.4	22.1 ± 4.7	26.9 ± 5.6	22.4 ± 5.2	27.0 ± 5.7	22.9 ± 5.6	24.0 ± 4.7
Total TV (mL)	26.6 ± 8.9	29.4 ± 6.7	27.6 ± 6.9	18.1 ± 9.2	7.9 ± 4.5	27.5 ± 8.5	24.0 ± 9.8
LH (UI/L)	4.1 ± 2.3	3.6 ± 1.7	3.2 ± 2.1	5.2 ± 2.8	8.6 ± 2.9	4.0 ± 2.0	4.5 ± 3.1
FSH (UI/L)	5.5 ± 5.9	3.4 ± 2.0	4.1 ± 2.6	11.7 ± 8.4	17.5 ± 10.2	4.6 ± 3.9	7.8 ± 9.2
TT (ng/mL)	4.5 ± 1.8	4.6 ± 1.9	5.4 ± 1.2	3.8 ± 1.2	3.2 ± 0.9	4.5 ± 1.8	4.5 ± 1.9
AMH (ng/mL)	5.7 ± 5.0	6.2 ± 6.3	/	3.9 ± 2.2	8.1 ± 2.3	6.5 ± 5.8	4.0 ± 1.3
Sperm concentration (10^6^/mL)	46.8 ± 45.3	52.0 ± 46.1	64.1 ± 46.3	12.5 ± 25.0	0 ± 0	48.2 ± 48.1	43.8 ± 40.8

**Table 2:** Clinical features of childhood cancer survivors according to diagnosis (mean ± DS). ALL: acute lymphoblastic leukemia; AMH: anti-Müllerian hormone; FSH: follicle-stimulating hormone; HD: Hodgkin’s disease; LH: luteinizing hormone; NHL: non-Hodgkin’s lymphoma; TT: total testosterone; TV: testicular volume; OT: other malignancies; WT: Wilms tumor. Reference values: TV ≥24 mL, LH 1.14–8.75 UI/L, FSH 0.95–11.95 UI/L, TT ≥3.5 ng/mL (gray zone 2.3–3.5 ng/mL), AMH 0.73–16.05 ng/mL, sperm concentration >39 × 10^6^/mL.

Clinical Features	ALL	NHL	HD	WT	OT
Age at diagnosis (years)	5.7 ± 3.9	6.8 ± 3.3	10.3 ± 4.1	2.4 ± 1.9	7.2 ± 4.9
Age at enrollment (years)	23.6 ± 5.7	22.3 ± 4.5	24.1 ± 5.4	22.4 ± 6.3	21.9 ± 5
Total TV (mL)	28.1 ± 8.1	23.8 ± 10	12.5 ± 3.5	28.8 ± 8.8	27 ± 9.5
LH (UI/L)	3.9 ± 2	4.2 ± 3.5	6.6 ± 2.8	4.2 ± 2.7	3.9 ± 2.5
FSH (UI/L)	4.1 ± 3.1	6.7 ± 7.1	15.8 ± 8.8	8 ± 9.4	6.2 ± 8.6
TT (ng/mL)	4.9 ± 1.9	3.7 ± 1.4	3.3 ± 0.7	5.2 ± 1	4.1 ± 1.2
AMH (ng/mL)	5.2 ± 2.6	23.2	5.4	2.3	2.9
Sperm concentration (10^6^/mL)	47.2 ± 36.5	52 ± 46.1	0 ± 0	/	68.8 ± 94.4
